# Tissue-Type Plasminogen Activator and Tenecteplase-Mediated Increase in Blood Brain Barrier Permeability Involves Cell Intrinsic Complement

**DOI:** 10.3389/fneur.2020.577272

**Published:** 2020-12-08

**Authors:** Charithani B. Keragala, Trent M. Woodruff, Zikou Liu, Be'eri Niego, Heidi Ho, Zoe McQuilten, Robert L. Medcalf

**Affiliations:** ^1^Molecular Neurotrauma and Haemostasis, Australian Centre for Blood Diseases, Monash University, Melbourne, VIC, Australia; ^2^School of Biomedical Sciences, University of Queensland, Brisbane, QLD, Australia; ^3^Transfusion Research Unit, Department of Epidemiology and Preventative Medicine, Australian and New Zealand Intensive Care Research Centre, Monash University, Melbourne, VIC, Australia

**Keywords:** fibrinolysis, plasminogen activation, complement, blood brain barrier, tissue-type plasminogen activator, tenecteplase

## Abstract

**Background:** Tissue-type plasminogen activator (t-PA) has been the mainstay of therapeutic thrombolysis for patients with acute ischaemic stroke (AIS). However, t-PA can cause devastating intracerebral hemorrhage. t-PA can also influence the CNS in part by modulation of BBB permeability. Complement activation also occurs after AIS and has also been reported to increase BBB permeability. The complement components, C3 and C5, can also be activated by t-PA via plasmin formation and cell intrinsic complement may be involved in this process. Tenecteplase (TNK-tPA) is a t-PA variant with a longer plasma half-life, yet the ability of TNK-tPA to modulate the BBB and complement is less clear.

**Aim:** To evaluate the effect of C5 and C5a-receptor 1 (C5aR1) inhibitors on t-PA- and TNK-tPA-mediated opening of the BBB.

**Methods:** We used an *in vitro* model of the BBB where human brain endothelial cells and human astrocytes were co-cultured on the opposite sides of a porous membrane assembled in transwell inserts. The luminal (endothelial) compartment was stimulated with t-PA or TNK-tPA together with plasminogen, in the presence of PMX205 (a non-competitive C5aR1 antagonist), Avacopan (a competitive C5aR1 antagonist) or Eculizumab (a humanized monoclonal inhibitor of human C5). BBB permeability was assessed 5 and 24 h later. Immunofluorescence was also used to detect changes in C5 and C5aR1 expression in endothelial cells and astrocytes.

**Results:** PMX205, but not Avacopan or Eculizumab, blocked t-PA-mediated increase in BBB permeability at both the 5 and 24 h time points. PMX205 also blocked TNK-tPA-mediated increase in BBB permeability. Immunofluorescence analysis revealed intracellular staining of C5 in both cell types. C5aR1 expression was also detected on the cell surfaces and also located intracellularly in both cell types.

**Conclusion:** t-PA and TNK-tPA-mediated increase in BBB permeability involves C5aR1 receptor activation from cell-derived C5a. Selective inhibitors of C5aR1 may have therapeutic potential in AIS.

## Introduction

The advent of recombinant tissue-type plasminogen activator (t-PA) revolutionized the management of acute ischaemic stroke more than two decades ago, providing a new therapeutic intervention for a devastating neurological disorder (1). However, tPA thrombolysis remains ineffective in achieving successful recanalization in 60% of the stringently selected patients (2). Furthermore, the risk of symptomatic intracerebral hemorrhage, the most feared and devastating complication of tPA thrombolysis, occurs in ~6.8% of cases (3). Despite the poor efficacy and risks, t-PA still remains the only approved pharmacological treatment available in patients with acute ischaemic stroke. While there have been no successful attempts to improve the efficacy of t-PA thrombolysis, there are also no treatments available to reduce the risk of t-PA induced ICH. Even in the current era of thrombectomy, tPA is still generally delivered in conjunction with mechanical clot retrieval. The need to minimize tPA's adverse effects while still harnessing its therapeutic potential remains a clinical and scientific priority.

Challenging tPA is tenecteplase (TNK-tPA), a t-PA variant developed over 25 years ago to circumvent the short (5 min) plasma half-life of t-PA. Only 6 amino acids are altered in TNK-tPA (hence is 99% identical to tPA), but these critical substitutions extended the plasma half-life of TNK-tPA to ~30 min. It is evident that any attempts to improve the efficacy and safety of t-PA should also include parallel studies on TNK-tPA in the event that TNK-tPA becomes the front line thrombolytic in the future.

The plasminogen activating system is traditionally known for its role in fibrinolysis. However, both tPA and plasmin have numerous non-fibrinolytic properties. In the CNS, plasmin and tPA are important in modulating NMDA receptors and synaptic plasticity, thus contributing to elements of learning and behavior (4, 5). tPA also has the ability to directly increase permeability of the blood brain barrier (BBB), which is considered the precursor to the development of intracerebral hemorrhage (6). Several plasmin-dependent and -independent mechanisms contributing to these permeability changes have been described (7–9). Intuitively, studies are exploring targeted inhibition of these pathways, aimed at reducing tPA's unwanted effects on the BBB while still maintaining its desired plasmin dependent fibrinolytic capacity.

Many signaling pathways are triggered either directly by t-PA or indirectly via plasmin generation that modulate permeability properties of brain microvascular endothelial cells or astrocytes that in turn impact on BBB integrity (6). Among these include the complement system that has received much attention in CNS disorders in recent years. The complement system, a crucial line of host innate immune defense, comprises a cascade of molecules which when activated, leads to cleavage of central components C3 and C5 to C3a and C5a, which then bind to their receptors (C3aR and C5aR1) and induce immune modulation. Furthermore, emerging literature recognizes the importance of intracellular complement components or the “complosome” in cell signaling repertoire (10). Whether this pathway is also involved in the tPA and plasmin mediated complement activation within astrocytes and other neuronal cell populations requires further investigation.

Complement's role in neuroinflammation, neurodegenerative disease and CNS injury is well-recognized (11–13). Plasmin's ability to directly activate complement proteins independent of the classical central C3 convertase, introduces a new paradigm to the understanding of complement activation (14). Recent studies have shown that tPA promotes C3 cleavage both *in vitro* and in the ischaemic mouse brain through a plasmin-mediated pathway (15). These authors also showed that the C3a-receptor is strongly expressed on the endothelium of ischaemic brain and that exogenous C3a dramatically enhanced brain endothelial cell permeability. tPA therapy was shown to exacerbate brain oedema and hemorrhage in stroke and these effects were ameliorated by treatment with a C3a receptor inhibitor (15). These findings established that intravenous tPA can upregulate complement activation in ischaemic brain tissue and that complement inhibition can protect against the adverse outcomes of tPA-mediated thrombolysis in stroke (15).

In contrast, the role of C5 and C5a in tPA-mediated neuroinflammation and stroke has not been explored. In relation to BBB integrity, Mahajan et al. demonstrated that C5a regulates BBB integrity during neuroinflammation, where it affects both endothelial and astroglial cells in a human *in vitro* model of systemic lupus erythematosus (SLE) (12). Furthermore, Eculizumab, a humanized monoclonal anti-C5 antibody approved for the treatment of the rare paroxysmal nocturnal haemoglobinuria (PNH) and atypical haemolytic uraemic syndrome (aHUS) was found to reduce the number of neurological episodes in neuromyelitis optica (NMO); a demyelinating disease characterized by BBB disruption and inflammation/degeneration of the optic nerve and spinal cord (16). Small molecule C5aR1 inhibitors (PMX205 and PMX53) have had some promising results in animal models of spinal cord injury (17), Alzheimer's disease (18), Amyotrophic lateral sclerosis (19, 20) and Huntington's disease (21, 22). C5aR1 deficient mice have also been reported to be protected in ischaemic stroke models, in a manner linked to neuronally derived (cell intrinsic) C5a (23). Avacopan, a clinically advanced small molecule C5aR1 antagonist, may have disease ameliorating and steroid sparing effects in ANCA associated vasculitis (24).

While tPA can increase BBB permeability and trigger the infiltration of immune cells into the CNS, complement activation may occur concomitantly with immune cell infiltration and has been linked to BBB permeability in other CNS injury models. Since plasmin is known to possess C3 and C5 convertase activity, we hypothesized that plasmin-driven complement activation is central to the capacity of the complement system to increase BBB permeability and inhibition of this pathway may offer a novel means to attenuate this process and abrogate the deleterious effects of thrombolytic therapy on the BBB and elsewhere in the brain.

Curiously, despite the fact that TNK-tPA has been available for over 25 years, it is still unclear whether or to what extent it shares any of these emerging non-canonical properties with tPA, particularly on the BBB and complement activation at the cellular level. Previous studies have reported that TNK-tPA can also increase BBB permeability in *in vitro* models but was ~50% as effective as tPA on a molar basis (25). TNK-tPA can also bind to NMDA subunits but whether it also influences neurological parameters to the same extent as tPA is unknown. In this study, we investigated the role of complement activation during t-PA and TNK-tPA-mediated opening of the BBB using an established *in vitro* model (26) using C5 and C5a-receptor 1 (C5aR1) inhibitors.

## Materials and Methods

### Reagents

Human t-PA (rt-PA; Actilyse®) was purchased from Boehringer Ingelheim GmbH (Rhein, Germany) and dialysed against 0.4 M HEPES at pH 7.4 to remove the original vehicle component from the compound. Tenecteplase was obtained from expired hospital stocks and kindly provided by Prof Christopher Levi (Hunter Valley Hospital, Newcastle, NSW, Australia). Human Glu-plasminogen was purchased from Enzyme research laboratories (South Bend, IN, USA). Fluorescein isothiocyanate- conjugated bovine serum albumin (FITC-BSA) was obtained from Sigma Aldrich (St Louis, MO, USA). Rat Collagen-I was purchased from Cultrex (Minneapolis, MN, USA).

PMX205 was synthesized and purified as previously described (27). Avacopan was purchased from MedKoo Biosciences, Morrisville, USA. Eculizumab remaining from therapeutic use was kindly donated by the Pharmacy department at Monash Health, Victoria, Australia.

### Institutional Ethics Approval

Normal human plasma used for the amidolytic assays was approved by the Local Institutional Human Research Ethics Committee, approval number 2017-9712-13995.

### Cell Culture

Primary human brain microvascular endothelial cells (BECs; ACBRI 376, Cell Systems, Kirkland, WA, USA) and human transformed SVG astrocytes were cultured to form an *in vitro* human BBB as described below. Endothelial Cell Growth Medium MV2 with Supplement Mix (Promocell, Heidelberg, Germany) was used as the BECs culturing growth medium. Minimum Essential Medium (MEM; Gibco, Thermo Fisher Scientific, Waltham, MA, USA) supplemented with 20% heat inactivated fetal calf serum, 1% L-Glutamine and 0.5% Penicillin/Streptomycin was used to culture the SVG astrocytes. BECs and SVGs were cultured for 3–4 days with a media change on day 3 and passage splitting on day 3 or 4.

Human monocytic leukaemic cells (THP-1) were cultured in RPMI supplemented with 10% heat inactivated fetal calf serum, serum, 1% L-Glutamine and 0.5% Penicillin/Streptomycin.

### *In vitro* Human Blood Brain Barrier Model

**Figure 1:**
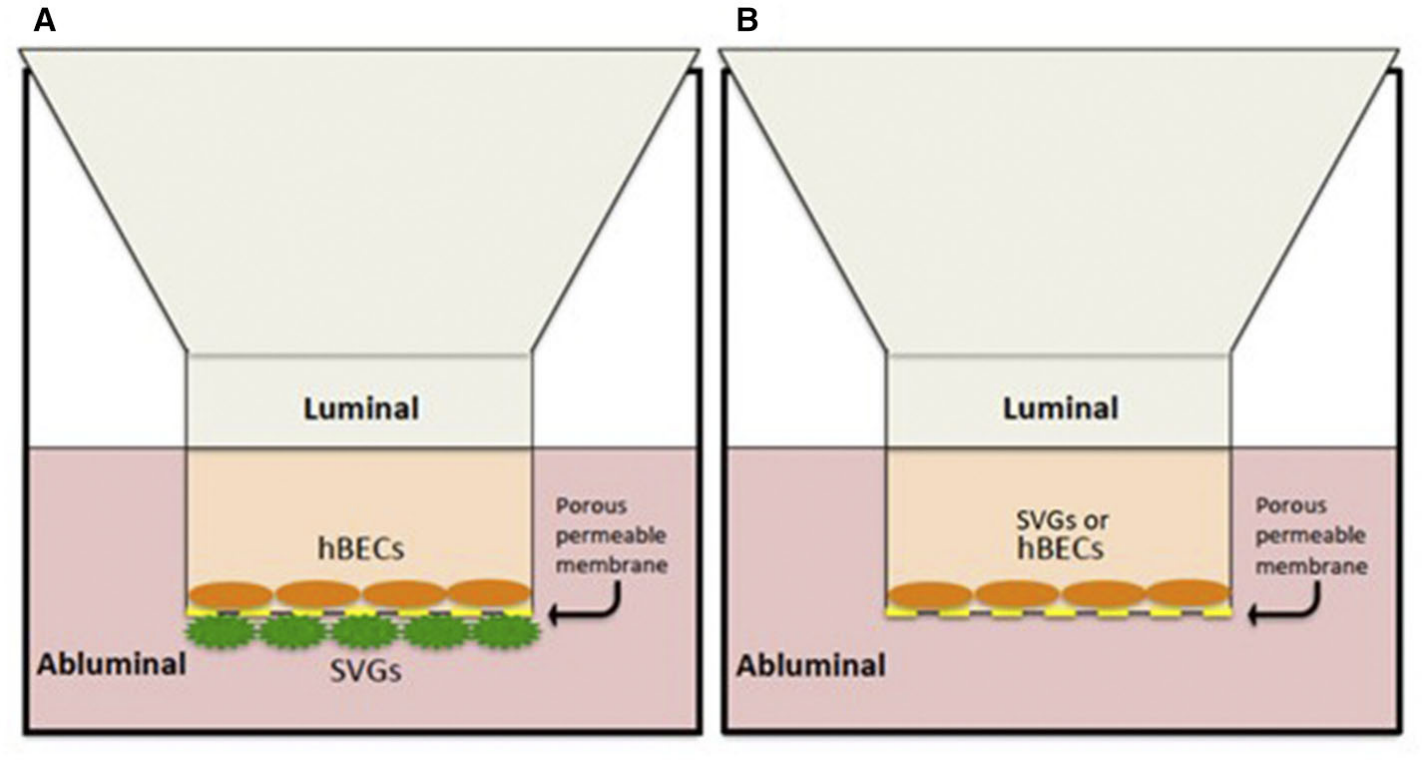
*In vitro* human BBB models. **(A)** Co-culture contact system with hBECs cultured on the luminal side of a porous membrane (0.4 μm pore size) and SVGs on the abluminal surface. **(B)** A non-contact monolayer of hBECs or SVGs cultured only on the luminal compartment.

An *in vitro* model of the BBB was utilized in line with our pre-existing protocol developed within our laboratory (25, 26) (Figure 1A). This involves co-culture of the BECs and SVGs on opposing surfaces of a porous, collagen-I-coated membrane using Transwell® inserts (polyester membrane, 6.5 mm diameter, 0.4 μm pore size, Costar, Corning, NY, USA).

Firstly, 4 × 10^4^ SVGs were allowed to adhere onto the underside of an inverted Transwell® insert over 3–4 h. Subsequently, 2 × 10^4^ BECs were cultured on the collagen-coated inner surface of the porous membrane on the same insert. This co-culture system was maintained in BEC growth medium over 3 days in a humidified 37°C incubator at 5% CO_2_ and 21% O_2_.

Experimentation on the BBB construct was undertaken after 3 days of incubation. Both luminal and abluminal chambers were washed once with serum free BEC medium not containing the Supplement Mix for 60 min. Thereafter, the luminal medium was replaced again with serum free BEC medium containing a cocktail of tPA (25 nM) or TNK-tPA (25 or 50 nM), plasminogen (100 nM) and the varied drugs of investigation at the specified concentrations. The investigational drugs, PMX205 (a cyclic peptide non-competitive C5aR1 antagonist) was reconstituted in sterile milliQ water and used at a final concentration of 10, 50, or 100 μM; Avacopan (a small molecule competitive C5aR1 antagonist) was reconstituted in 100% dimethyl sulfoxide (DMSO) and used at 2 μM; and Eculizumab, a humanized monoclonal inhibitor of human C5, was supplied as a 10 mg/ml aqueous solution and used at a final concentration of 200 μg/ml (1.35 μM). These reagents were added to both endothelial (luminal) and astrocytic (abluminal) compartments simultaneously. tPA, TNK-tPA and plasminogen were only added to the luminal/endothelial compartments. We chose doses of PMX205 and Avacopan based on the known pharmacological properties of these compounds in human cells (28, 29). Eculizumab was used at a 1:50 final dilution of the 10 mg/ml stock solution (200 μg/ml) and approximates the concentration used clinically for the treatment of patients with PNH (30). In experiments where PMX205 was evaluated on single cell cultures of BECs or SVG astrocytes, it was added to the luminal compartment only.

Incubation durations were either 5 or 24 h. Changes in the BBB permeability were evaluated by adding fluorescent FITC-conjugated BSA 0.385% (w/v) to the luminal chamber 1 h prior to the end point (i.e., at 4 or 23 h post treatment) and sampling the abluminal chamber 1 h later to quantitate the passage of the tracer through the semipermeable BBB over this 1 h period. Fluorescence was measured using a micro-plate reader (FLUOstar Optima, BMG labtech, Australia) at an emission wavelength of 485 nm and absorbance wavelength of 520 nm and gain adjusted to 1,500 nm. Data is presented as a fold-change in the passage of FITC-BSA relative to vehicle treated control wells.

It is important to note that endothelial tight junction proteins like ZO-1 are integral components of blood brain barrier integrity. Using immunofluorescence, we have previously reported the presence of ZO-1 along BEC-BEC boundaries (31). Furthermore, a breakdown of tight-junction proteins in BECs by t-PA and plasminogen and their preservation by a ROCK-2 selective inhibitor (KD025) became apparent, fully correlating with the permeability data. In addition, we confirmed that the ability of t-PA to modulate permeability was not due to any toxic effects on cell viability but rather due to direct changes on cell signaling events. These results indicate the involvement of tight junction proteins in our experimental system further supporting their utility as a model for the BBB.

A “non-contact” monolayer *in vitro* system was also utilized to isolate and identify any individual contribution from each cell type (BECs or SVGs) to the BBB changes seen (Figure 1B). This involved a simplified version of the co-culture system where only the luminal chamber of the inserts was cultured with BECs or SVGs and no cells were cultured on the abluminal surface of the porous membrane. The stimulation “cocktail” was added only to the luminal compartment and evaluation of changes to the cell layer was performed in the same manner as described for the co-culture system.

### Amidolytic Chromogenic Assay for Plasmin Generation

Plasmin activity/activation was evaluated using an amidolytic assay with S-2251 (Chromogenix S-2251, Diapharma, Louisville, USA) a chromogenic substrate specific for plasmin. This was performed to exclude any confounding anti-plasmin properties of the complement inhibitors under investigation. Aprotinin (Sigma-Aldrich, Missouri, USA), a serine protease inhibitor that competitively inhibits plasmin was used as a positive control (32). This assay has previously been optimized by our laboratory to evaluate the plasmin generation rate in plasma (33). t-PA (30 nM) in the presence of a cofactor (cyanogen bromide activated (CNBr) fibrinogen at 0.25 mg/mL) and S2251 (1 mM) “spiked” with complement inhibitors PMX205 (10, 100 μM) and Avacopan (2 μM) was incubated with healthy donor control plasma in a clear 96-well plate and absorbance read every cycle (20 s cycle) at 405 nm for 250 cycles using a plate reader pre-set at 37°C. Recordings of the optical densities taken every 20 s was collated and a sigmoidal curve generated. The curves exhibit an initial lag phase, followed by an exponential growth phase reflecting plasmin generation in the sample and a final plateau phase indicating exhaustion of the S2251 substrate.

### Phase Contrast Microscopy and Immunofluorescence Confocal Imaging

BECs and SVGs were cultured to confluency over 3–4 days in 12-well plates (for phase contrast imaging) or in μ-slides 8-wells (Ibidi, Munic, Germany) for immunofluorescence. For phase contrast microscopy, wells were coated with gelatine for BECs and treated with serum free medium containing t-PA (25 nM) and plasminogen (100 nM) with or without PMX205, Avacopan or Eculizumab at the same concentrations used in the *in vitro* system. Stimulation times were ~15–20 h in duration. Following aspiration of the stimulation media, the cells were gently washed with PBS then fixed for 30 min with ice-cold 4% paraformaldehyde (PFA). Phase contrast images were captured using a Nikon Eclipse TS100 inverted microscope.

For immunofluorescence imaging, cells cultured in the μ-slides were treated with serum free medium alone or containing t-PA (25 nM) and plasminogen (100 nM) for a period of 15–20 h. Following aspiration of the stimulation media the cells were fixed with ice-cold 4% PFA for 10 min. Thereafter, the cells were blocked for 10 min with TBS containing 10% heat inactivated horse serum. Following 3 × 5 min washes with TBS, the primary antibody was added and incubated overnight at 4°C with gentle agitation. The primary antibodies used include mouse anti-human C5aR (CD88), clone W17/1 (HycultBiotech, Netherlands) diluted 1:50 and sheep anti-human C5 (Bio-Rad, California, USA) diluted 1:100. Secondary antibodies used include Alexa Fluor 568-conjugated donkey anti-mouse and Alexa Fluor 488-conjugated chicken anti-mouse (Invitrogen, California, USA) incubated for 3 h at 4°C with gentle agitation, diluted 1:1,000. DyLight488-conjugated donkey anti sheep/goat IgG (Bio-Rad, California, USA) was used as the paired secondary to the anti-human C5 primary antibody, diluted 1:100 as per the manufacturer's recommendations. All primary and secondary antibodies were diluted in TBS containing 4% heat inactivated horse serum. Hoechst was used as a nuclear counterstain (Invitrogen, California, USA) at a final concentration of 5 μg/mL in TBS, incubated for 30 min at 4°C with gentle agitation. Immunofluorescence images were captured on a Nikon A1r confocal inverted microscope using a X40 water objective and fluorescence DAPI, FITC/Alexa488 and Alexa 568. Nikon NIS-Elements software was used to directly acquire the images and further analysis was completed using FIJI (Image J) software.

THP-1 cells were used as a positive control for C5aR and C5 immunofluorescence staining. These non-adherent cells were cultured and adhered to a glass slide using a cytospin centrifuge (1,000 rpm, low acceleration, 5 min). Two-hundred microliter of cell stock at a concentration of 100,000 cells/mL was used for each slide. The cells were fixed and stained alongside the μ-slides containing BECs and SVGs as per the protocol described above.

### Statistical Analysis

Each *in vitro* BBB experiment was performed in triplicate and at least four independent experiments were performed. Statistical analysis was performed using GraphPad Prism 8.0 software. Comparisons of experimental data sets were performed by ordinary one-way ANOVA with the application of the Dunnett's multiple comparisons test. *P*-values under 0.05 were considered significant.

## Results

### Non-competitive C5aR1 Antagonist, PMX205, Reduces t-PA-Mediated Increase in BBB Permeability

To determine whether the t-PA/plasmin-mediated increase in BBB permeability could be inhibited by targeting the complement system, we evaluated the capacity of three different inhibitors of the complement pathway: PMX205 (cyclic peptide non-competitive C5aR1 antagonist), Avacopan (a small molecule competitive C5aR1 antagonist) and Eculizumab (a humanized monoclonal inhibitor of human C5) to attenuate the actions of tPA/plasmin in our established *in vitro* BBB model.

**Figure 2:**
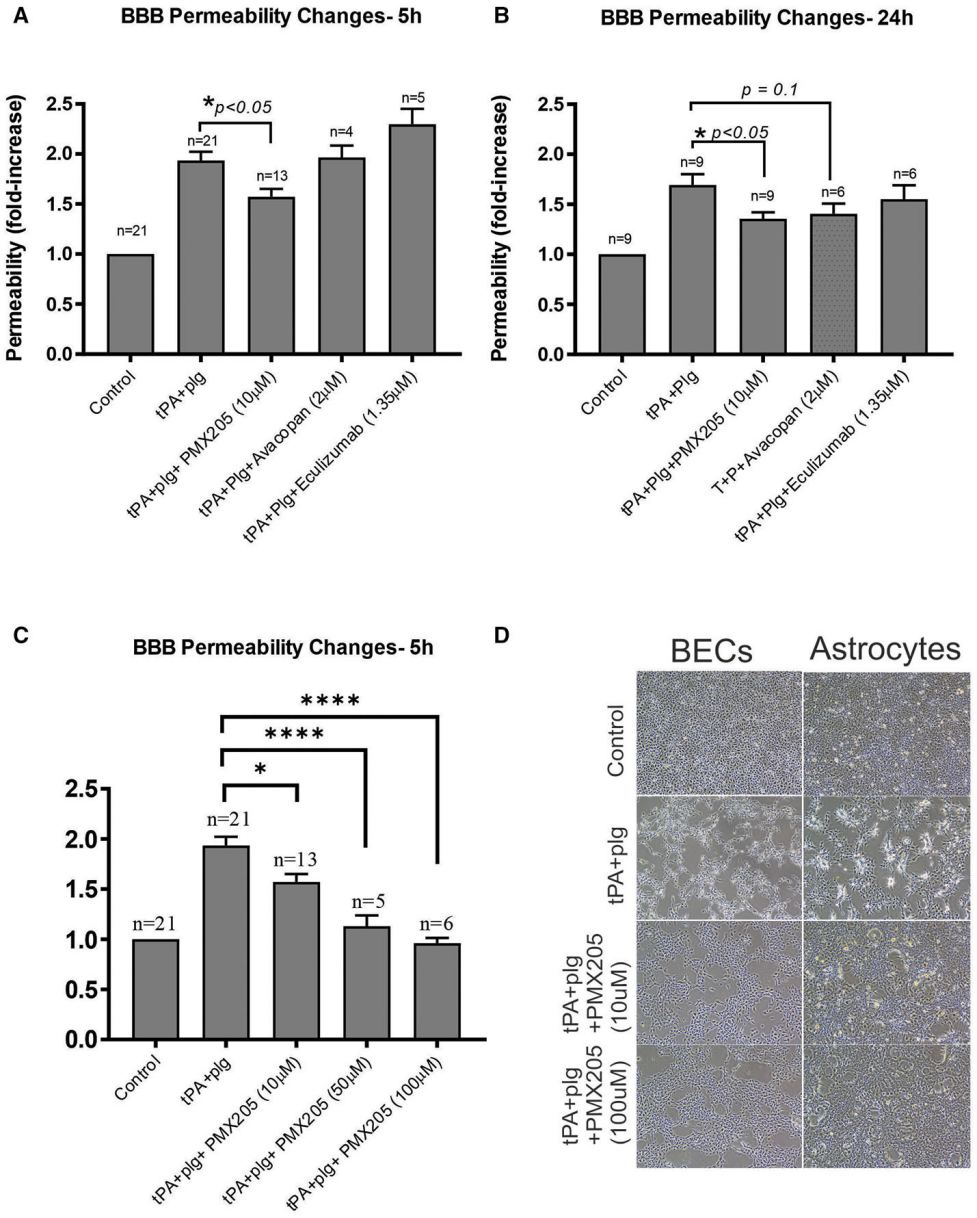
The small molecule C5aR1 inhibitor, PMX205, reduces the t-PA and Plg-mediated increase in BBB permeability at 5 and 24 h. **(A)** Permeability changes in the *in vitro* human BBB 5 h post stimulation with control (serum-free medium) or with t-PA (25 nM) and human Plg (100 nM), with or without PMX205, Avacopan or Eculizumab (10, 2, or 1.35 μM, respectively, added to both luminal and abluminal chambers) **(B)** Permeability changes at 24 h post-stimulation with control (serum-free medium) or with t-PA (25 nM) and human Plg (100 nM), with or without PMX205, Avacopan or Eculizumab at same concentration and protocol as per 5 h experiment. **(C)** Permeability changes in the *in vitro* BBB 5 h post-stimulation with control (serum-free medium) or with t-PA (25 nM) and human Plg (100 nM), with or without PMX205 at increasing concentrations (10, 50, 100 μM, added to both luminal and abluminal chambers). **(D)** Phase-contrast microscopy images (x 4 magnification) of BECs and SVG human astrocytes demonstrating blockade of morphological changes by PMX205 at ~20 h of treatment compared with control (serum-free medium), or t-PA (25 nM) and Plg (100 nM), in the presence or absence of PMX205 (10 or 100 μM). Data is presented relative to serum-free medium control. Bars represent mean ± SEM. *p < 0.05, ****p < 0.0005 compared to t-PA+Plg by one-way ANOVA with Dunnett's multiple comparison test.

PMX205, significantly reduced t-PA+plasminogen (plg)-mediated increase in BBB permeability at both 5 h (Figure 2A) and 24 h (Figure 2B) after treatment. This effect of PMX205 was dose-dependent (Figure 2C). Indeed, the increase in BBB permeability was reduced by 36% at 5 h (*p* < 0.05) and 34% at 24 h (*p* < 0.05) using a 10 μM concentration. This increased to an 80% reduction in permeability with the higher 50 μM dose (*p* < 0.0001) and 97% using 100 μM of PMX205 (*P* < 0.0001) both at the 5 h time point.

Interestingly, unlike PMX205, Avacopan, had no effect on BBB permeability at the 5 h time point (Figure 2A), but showed a non-significant (*P* = 0.1) 29% reduction in this increased permeability at 24 h (Figure 2B). Eculizumab had no significant effect at either time point (Figures 2A,B).

We have previously reported that t-PA promotes marked morphological changes of both BECS and astrocytes by altering contractility of the cytoskeletal structure of the cells via the Rho-kinase (ROCK) pathway (25). As shown in Figure 2D, the capacity of t-PA+plg to alter cell morphology and contractility of both cell types was blocked by PMX205 in a dose-dependent manner as revealed by phase contrast microscopy.

### BECs Contribute the Majority of the BBB Permeability Changes to C5aR1 Inhibition With PMX205 Compared to Astrocytes

**Figure 3:**
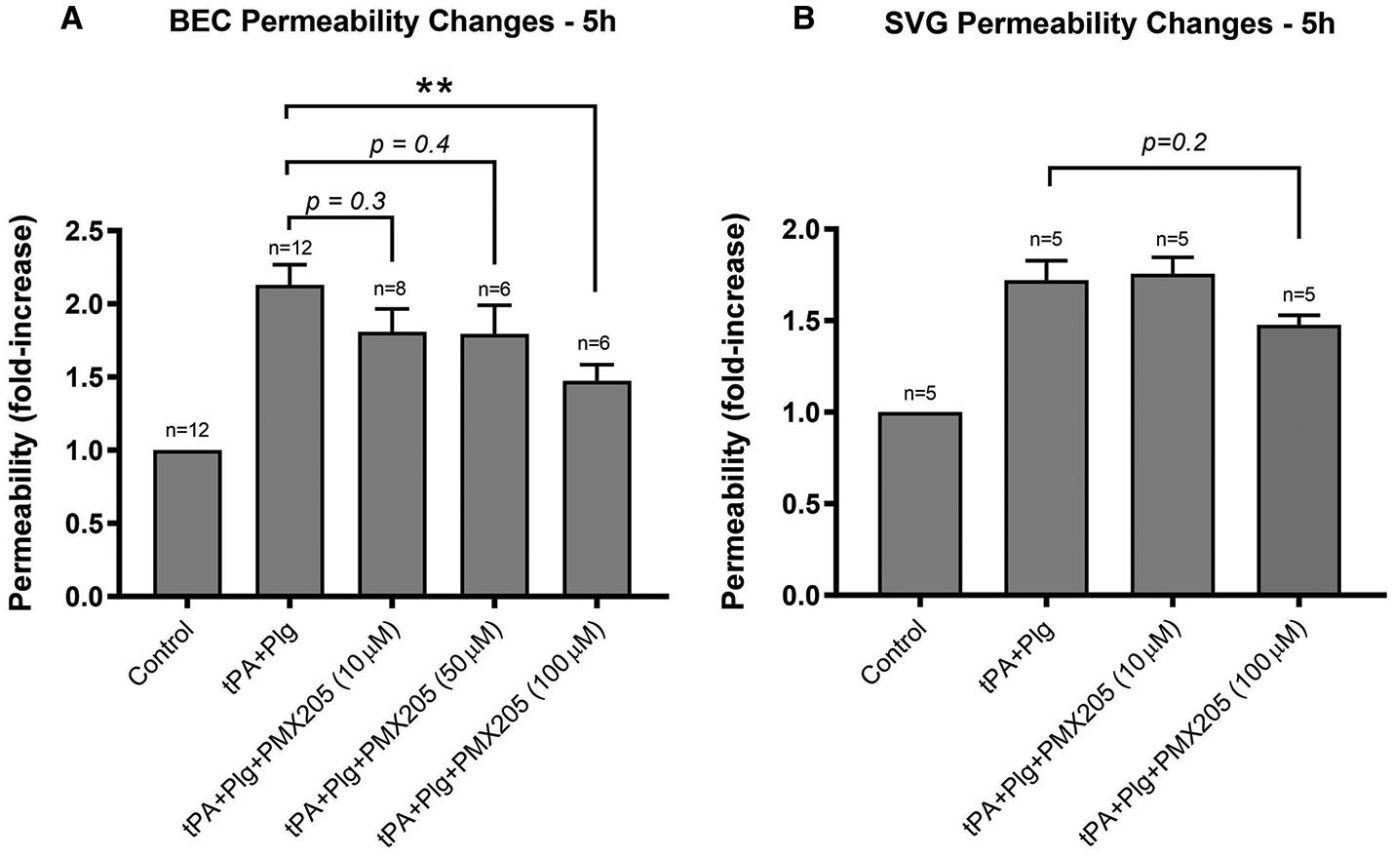
BECs contributed the majority of the BBB permeability changes to C5aR1 inhibition with PMX205 compared to SVG human astrocytes. **(A)** Permeability changes in BEC monolayers alone 5 h post-stimulation with control (serum-free medium) or with t-PA (25 nM) and human plasminogen (plg; 100 nM), with or without PMX205 (10, 50, or 100 μM added only to the luminal chamber) **(B)** Monolayer integrity changes in SVG astrocytes cultured alone 5 h post-stimulation with control (serum-free medium) or with t-PA (25 nM) and human Plg (100 nM), with or without PMX205 (10 or 100 μM added only to the luminal chamber). Data is presented relative to serum-free medium control. Bars represent mean ± SEM. **p < 0.005, compared to t-PA+Plg by one-way ANOVA with Dunnett's multiple comparison test.

We next investigated whether this complement inhibitory effect on the BBB was mediated by one particular cellular compartment more than the other. A single cell monolayer BBB model was used to stimulate BECs and SVGs individually with the same stimulation protocol used in the contact (co-culture) system. Both BECs and SVGs, when cultured alone, showed a significant increase in cell permeability following treatment with tPA and plasminogen (Figures 3A,B), but this was more marked in BECs (2.13 ± 0.48-fold increase) than astrocytes (1.72 ± 0.32-fold increase), suggesting that BECs are more sensitive to tPA/plasmin. Interestingly, PMX205 reduced the capacity of tPA to increase BEC permeability, with the 100 μM dose significantly reducing permeability by 66% (*p* < 0.005; Figure 3A). In contrast, SVGs did not show a significant reduction in permeability fold change in the presence of C5aR1 inhibition (Figure 3B), even using the higher dose of 100 μM, although there was a trend toward a benefit.

### Small Molecule C5aR1 Inhibitors Do Not Exhibit Any Antiplasmin Properties

**Figure 4:**
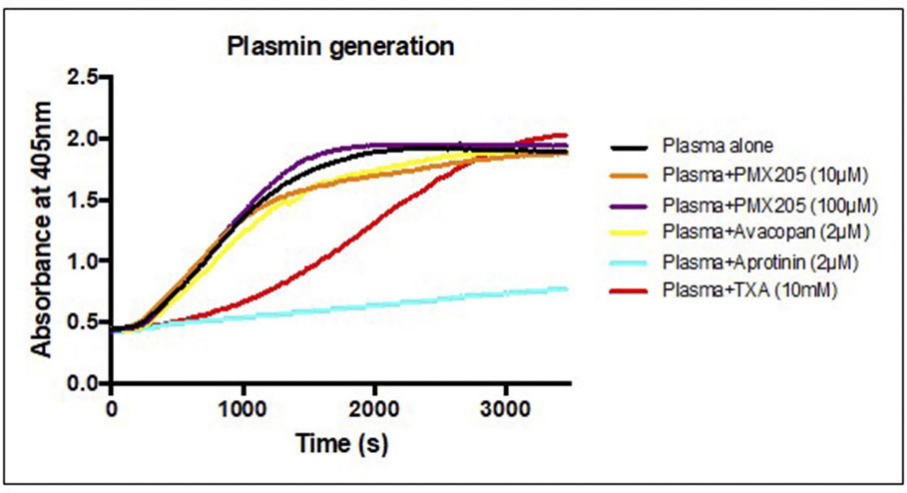
C5aR1 inhibitors have no direct plasmin inhibitory effect. S2251 plasmin generation curve of plasma from healthy subjects in the presence or absence of PMX205 (10, 100 μM), Avacopan (2 μM), Aprotinin (2 μM) or TXA (10 mM). Cleavage of the plasmin-specific chromogenic substrate S2251 was measured by the absorbance of 405 nm.

The *in vitro* results of the small molecule C5aR1 inhibitors, in particular PMX205, in ameliorating the t-PA and plasminogen- mediated permeability changes to the BBB raised the question of whether off-target direct anti-plasmin effects of these agents could be contributing to the observed reductions in BBB permeability. To address this question, we utilized the S2251 amidolytic assay that is specific for plasmin, which allowed us to evaluate effects of these complement inhibitors on plasmin activity. Normal healthy human control plasma was incubated with t-PA and S2251 in the presence and absence of PMX205 or Avacopan (see Methods). Results indicated there is minimal to absent anti-plasmin effects of PMX205 at 10 or 100 μM concentrations. Similarly, Avacopan at 2 μM did not show any anti-plasmin activity (Figure 4). Aprotinin (2 μM), a competitive inhibitor of plasmin was used as a positive control and showed almost complete plasmin inhibition. Furthermore, Tranexamic acid, a lysine analog, which impedes plasminogen and plasmin binding to their active sites significantly slowed down the plasmin generation curve.

### Immunofluorescence Imaging Reveals C5 and C5aR Expression in BECs and SVGs

The functional *in vitro* changes we observed in the absence of any serum-derived complement, prompted us to investigate the distribution of complement C5a receptor 1 and its ligand, C5, in BECs and SVGs, and their changes following t-PA and plasminogen treatment of these cells. Confocal immunofluorescence images of BECs and SVGs were obtained following co-staining for the C5aR1 (CD88) and C5. Human monocytic leukaemic cells (THP-1) were used as a positive control for C5aR1 and C5 (not shown) as these malignant cells are known to contain high levels of complement components (34).

**Figure 5:**
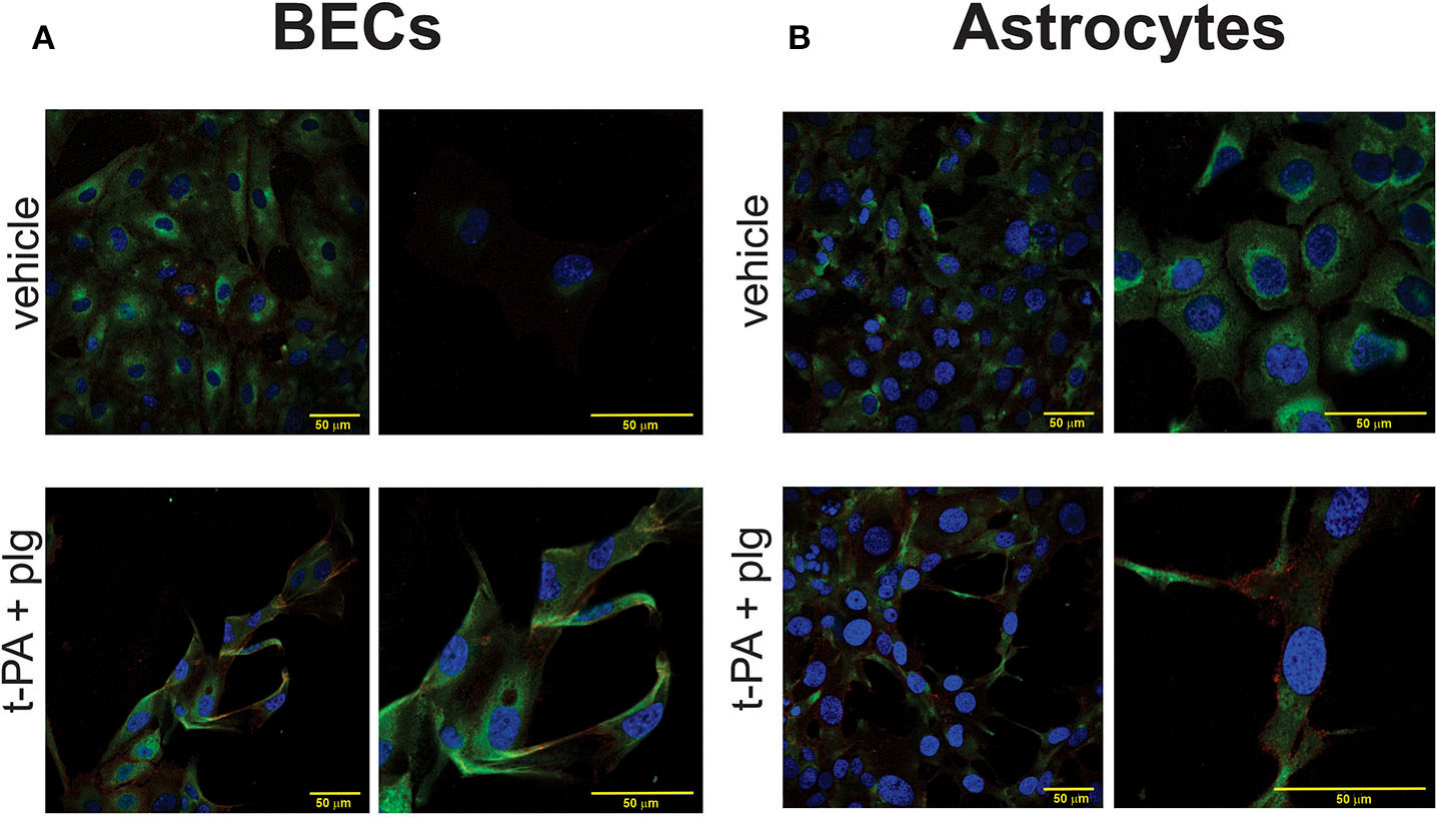
C5 and C5aR1 are expressed in BECs and SVG astrocytes at baseline and following treatment with t-PA and plasminogen. Representative confocal immunofluorescence images of C5 (green), C5aR1 (red) and nuclei (Hoechst; blue) of BECs **(A)** and SVG astrocytes **(B)**, demonstrating a prominence of C5 and C5aR1 in vehicle-treated cells and following treatment with t-PA (25 nM) and plg (100 nM) for ~20 h. Magnification scale bars are indicated in each panel.

The vehicle treated BECs exhibited a prominence of C5 signal intracellularly in the cytoplasm with an increased intensity closer to the perinuclear areas. A scattered low intensity signal for C5aR1 was evident predominantly in the cell periphery, with some distribution intracellularly in the cell cytoplasm. Vehicle treated SVGs also showed a similar pattern of distribution of the C5aR1 and C5 at baseline (Figures 5A,B). Following treatment with t-PA and plasminogen for approximately 20 h, the BECs exhibited a persistence in C5 signal which was again concentrated predominantly intracellularly, however this time there was distribution throughout the entire cell cytoplasm. The C5aR1 signal was also present following t-PA and plasminogen treatment, especially in the cell periphery and in some areas, delineating the cell outline quite clearly (Figure 5) suggestive of cell membrane expression. In addition to undergoing prominent cytoskeletal changes, the SVGs also showed prominent C5 and C5aR1 expression following t-PA and plasminogen treatment (Figure 5B). These immunofluorescence image findings indicate there is C5 and C5aR1 expression in untreated fixed BECs and SVGs and this signal persists following t-PA and plasminogen treatment. Although there appears to be a qualitative prominence of C5 and C5aR1 signal in the t-PA and plasminogen treated cells, quantitative studies, such as qPCR or flow cytometry, are needed to confirm any true upregulation of C5 and C5aR1 in this context.

### Delayed Administration of PMX205 Still Ameliorates the t-PA and Plasminogen Mediated Increase in BBB Permeability *in vitro*

**Figure 6:**
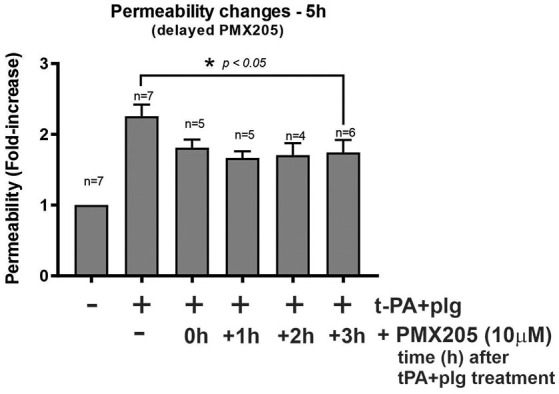
Delayed administration of PMX205 ameliorates the t-PA and plasminogen (plg)-mediated permeability increase of the *in vitro* BBB. Permeability changes in the *in vitro* human BBB 5 h post-stimulation with control (serum-free medium) or with t-PA (25 nM) and human plg (100 nM), with or without PMX205 10 μM added either 1 h prior to t-PA+Plg, or 1, 2, or 3 h after t-PA+Plg. Bars = SEM.

Given the results of PMX205 reducing t-PA and Plg-mediated increase in BBB permeability *in vitro* and the cellular prominence of C5aR1 and its ligand, C5, on immunofluorescence imaging, we were keen to understand whether PMX205 could reduce this increase in permeability at delayed timepoints following t-PA and Plg treatment of the BBB. We found the effects of PMX205 were still evident when drug was added up to 3 h after t-PA and Plg treatment (Figure 6). Interestingly, there did not appear to be a significant difference in outcome between early treatment with PMX205 (prior to t-PA and Plg) and the delayed administration of PMX205 post-t-PA and Plg.

### Tenecteplase Mediated Increase in BBB Permeability Is Also Inhibited by PMX205

**Figure 7:**
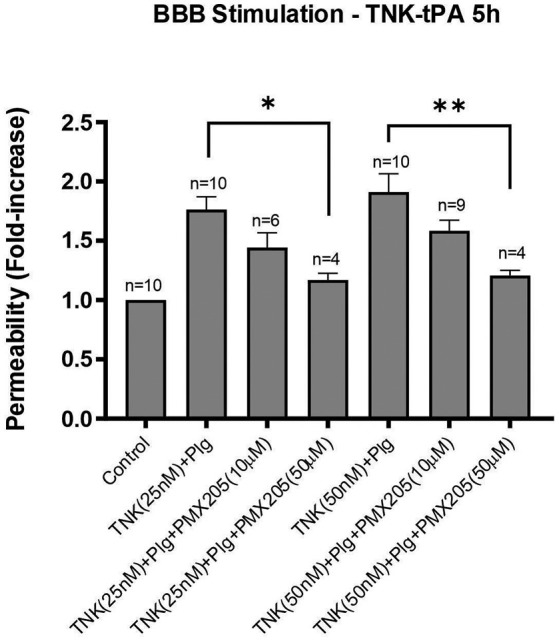
TNK-tPA mediated increase in BBB permeability is blocked by PMX205. Permeability changes in the *in vitro* human BBB 5 h post-stimulation with control (serum-free medium) or with TNK (25 or 50 nM) and human plasminogen (plg; 100 nM), with or without PMX205 (10 or 50 μM). PMX205 was added to both the luminal and abluminal chambers. Bars represent mean ± SEM. *p < 0.05, **p < 0.005, compared to TNK+Plg one-way ANOVA with Dunnett's multiple comparison test.

TNK treatment of the *in vitro* system resulted in a consistent increase in the BBB permeability both at 25 and 50 nM concentrations, although this was less potent than t-PA which supports previous reports (25). Nonetheless, TNK-tPA-mediated increase in BBB permeability was effectively inhibited by PMX205, although more prominently at the higher dosage of 50 μM. PMX205 treatment at 50 μM resulted in a 59% reduction in BBB permeability induced by TNK at 25 nM (*p* < 0.05) and a more impressive 70% reduction in BBB permeability induced by TNK at 50 nM (*p* < 0.005) (Figure 7).

## Discussion

The blood-brain barrier and the complement system are both critical for brain defense and homeostasis. Loss of BBB integrity and unregulated complement activation can have deleterious consequences in the CNS (35). This is most notable in ischaemic stroke where BBB breakdown (6) and complement activation (36) occur hand-in-hand. Although well known for its role in innate immunity, the defensive function of complement can actually exacerbate immune and inflammatory conditions (37) and complement inhibition has been reported to be protective in models of ischaemic stroke (38–40). Adding to this is the observation that complement activation can itself increase BBB permeability (12) further indicating that both processes are intertwined.

In this study, we have evaluated the role of the complement components C5a/C5aR1 during t-PA-mediated opening of the BBB using an *in vitro* model. This model had been previously validated as a relevant *in vitro* BBB model with the expression of the tight junction protein ZO-1 expressed between BEC-BEC boundaries. Our investigation into the role of complement was undertaken not only because the complement pathway is known to modulate the BBB (12), but that the key complement components C3 and C5 can be directly activated by t-PA via plasmin generation (41, 42). Hence, we took the view that the activation of these key complement components in patients with acute ischaemic stroke may be further facilitated by t-PA, providing an additional means by which t-PA can increase BBB permeability and intracellular bleeding. Hence, the role of plasmin-mediated complement activation in this landscape introduces an intriguing avenue contributing to the inflammatory and cellular changes seen within the BBB following t-PA exposure. This could potentially be a key event during t-PA thrombolysis when t-PA levels are transiently increased many hundred-fold leading to excessive plasmin-mediated cleavage of C5 and C3 that could not only exacerbate inflammation, but also increase BBB permeability and intracerebral bleeding.

The effects of tPA/plasmin-mediated C3 cleavage and its impact on the ischaemic brain have previously been explored (15). However, since the role of C5/C5a is yet to be investigated in this particular context, we set out to determine the extent to which C5/C5a participated in the ability of t-PA to increase BBB permeability. To address this, we compared the inhibitory capacity of three distinct C5 and C5aR1 blocking agents using an *in vitro* BBB model previously used in our laboratory, to study BBB permeability changes following t-PA and plasminogen treatment (25, 31). Tenecteplase (TNK-tPA) is closely related to t-PA and under development as a thrombolytic for AIS. However, there is little information available as to the extent to which TNK-tPA can modulate the BBB and whether TNK-tPA can also trigger complement activation. Hence, we were interested to compare and contrast t-PA with TNK-tPA in this study.

We showed that t-PA- and TNK-tPA-mediated increase in BBB permeability was inhibited using the small molecule non-competitive C5aR1 antagonist, PMX205. However, neither Avapocan (competitive C5aR1 antagonist) nor Eculizumab (monoclonal human C5 inhibitor) produced any significant inhibitory activity against t-PA mediated increase in BBB. We have previously shown that brain endothelial cells and astrocytes undergo significant cytoskeletal and morphological changes following exposure to tPA and plasminogen, a feature that is dependent in part, on activation of the Rho-kinase pathway (25). Since PMX205 also blocked the morphological effect of t-PA, this suggests that activation of C5 by t-PA/plasmin occurs upstream of the pathways controlling cytoskeletal changes in the cells forming the BBB, although this requires further investigation. Previous studies have reported on the specificity of PMX205 for C5aR1 and this inhibitor does not inhibit closely related receptors (i.e., C5ar2, C3aR, FPR1), and is inactive in C5aR1^−/−^ mice (43), suggesting the effects observed in our study were truly C5aR1-dependent. We also excluded the possibility that PMX205 was not acting directly on plasmin activity since addition of PMX205 had no effect on the amidolytic activity of plasmin.

We observed that BECs appeared to be the most sensitive to tPA and plasminogen-mediated cytoskeletal changes, reflected in the robust increase in monolayer permeability. Moreover, the blocking capacity of PMX205 seen in the BBB model, appeared to predominantly target BECs, as PMX205 failed to block that ability of t-PA to increase BBB permeability of the SVG astrocytes. Since both cell types displayed increased cell permeability in response to t-PA + plasminogen treatment, yet only the increase in BEC permeability was blocked by PMX205, indicates an important cell-type specific response to this inhibitor. On the other hand, PMX205 did partially reduce the ability of t-PA to promote morphological changes to the astrocytes. This may reflect differences in the level of C5a generated in BECs and astrocytes, but this remains to be determined.

In keeping with the response of the cells to PMX205, we observed cellular expression of C5aR1 in both BECs and SVGs at baseline and following t-PA and plasminogen exposure, as revealed by immunofluorescence. Prominent cytoplasmic staining was seen for both C5aR1 and C5 (especially C5) as well as along the cell periphery, at baseline and following t-PA exposure. It remains to be determined whether treatment of either cell type with t-PA+plasminogen further increases expression of these complement components as quantitative experiments are yet to be performed. Nonetheless, the intracellular presence of C5 and to some extent, C5aR1, is in contrast to the classical thinking that C5aR1 is solely a G-protein coupled transmembrane receptor. Indeed, our findings are more in keeping with recent literature suggesting that many of the complement components are expressed intracellularly within immune and non-immune cells, contributing to the entity coined the “complosome” which has important roles in cellular regulation and signaling (10, 44). Intracellular complement protein stores of C3 and C5 can be cleaved intracellularly or on the cell surface and also undergo binding to their respective intracellular receptors, C3aR and C5aR1 (44). We hypothesize that similar processes are playing a role in non-immune cells, such as astrocytes and endothelial cells used in our studies.

We were surprised that of the three complement inhibitors evaluated, only PMX205 proved to be effective at blocking BBB permeability. The prominent intracellular location of C5 probably explains why Eculizumab, a ~180 kD antibody molecule was ineffective at blocking the ability of t-PA to increase BBB permeability due to its inability to gain intracellular access. This result also suggests that cells are generating C5a intracellularly (i.e., not secreting C5 to be cleaved extracellularly) in response to t-PA treatment, which is either binding to intracellular or cell surface expressed C5aR1. While Avacopan, a small molecule (580 Da) had no demonstrable blocking effect on the BBB, we did observe that Avacopan did partially block the t-PA induced morphological changes in both BECs and in SVG astrocytes (data not shown), suggesting there was partial activity. Despite Avacopan being much more potent than PMX205 in other classical immune cell-based assays (45), it may be that higher doses of this compound were needed to observe full functional activity in our model system (and indeed perhaps to achieve intracellular C5aR1 inhibition).

PMX205 also blocked the ability of TNK-tPA to increase BBB permeability, most likely reflective of TNK-tPA-mediated plasmin generation that in turn activates C5. TNK-tPA was less effective than t-PA at increasing permeability overall which is consistent with our previous study (25). Nonetheless, patients treated with TNK-tPA are still at risk of developing symptomatic intracranial hemorrhage so the ability of PMX205 to inhibit this capacity of TNK-tPA also has potential therapeutic implications if TNK-tPA is to become the preferred thrombolytic for AIS.

Another key finding of our study was that delayed addition of PMX205 still effectively prevented t-PA-induced increases in BBB permeability when added up to 3 h after t-PA treatment. In fact, later addition of PMX205 seemed to be slightly more effective compared to the earlier time points, perhaps reflecting PMX205 activity on the upregulated C5aR1 at this timepoint. We have previously reported that the ability of tPA to increase BBB permeability could also be blocked by delayed addition of the plasmin inhibitor, aprotinin (46) over a similar time frame, supporting the notion that the process is reversible. This finding has significant positive clinical implications. If this holds true, delayed complement inhibition could serve as an adjunctive therapy following thrombolysis in acute ischaemic stroke or as part of supportive care in traumatic brain injury to limit the unwanted BBB effects of excess t-PA in these time critical contexts.

In conclusion, our data demonstrate that inhibition of complement C5a-C5aR1 interaction reduces the ability of both t-PA or TNK-tPA to increase BBB permeability, and may offer a novel means to improve the safety profile of thrombolytic therapy for patients with acute ischaemic stroke.

## Data Availability Statement

The raw data supporting the conclusions of this article will be made available by the authors, without undue reservation.

## Ethics Statement

The studies involving human participants were reviewed and approved by Monash University Human Research Ethics Committee. The patients/participants provided their written informed consent to participate in this study.

## Author Contributions

RM and CK: project concept and writing of the manuscript. CK, HH, and ZL: experimentation. CK, RM, and TW: data analysis. TW, ZM, and BN: intellectual input and project concept. All authors contributed to the article and approved the submitted version.

## Conflict of Interest

TW has previously consulted to Alsonex Pty Ltd., who are commercially developing PMX205, and Alexion Pharmaceuticals Inc., who developed eculizumab. He holds no stocks, shares or other commercial interest in either company. The remaining authors declare that the research was conducted in the absence of any commercial or financial relationships that could be construed as a potential conflict of interest.
